# Medically Unexplained Symptoms and Attachment Theory: The BodyMind Approach®

**DOI:** 10.3389/fpsyg.2019.01818

**Published:** 2019-11-06

**Authors:** Helen Payne, Susan D. Brooks

**Affiliations:** School of Education, University of Hertfordshire, Hertfordshire, United Kingdom

**Keywords:** medically unexplained symptoms, the bodymind approach, insecure attachment style, safety, self management intervention, group, facilitator, bodymindfulness

## Abstract

This article discusses how The BodyMind Approach^®^ (TBMA) addresses insecure attachment styles in medically unexplained symptoms (MUS). Insecure attachment styles are associated with adverse childhood experiences (ACEs) and MUS (Adshead and Guthrie, 2015) and affect sufferers’ capacity to self-manage. The article goes on to make a new hypothesis to account for TBMA’s effectiveness (Payne and Brooks, 2017), that is, it addresses insecure attachment styles, which may be present in some MUS sufferers, leading to their capacity to self-manage. Three insecure attachment styles (dismissive, pre-occupied and fearful) associated with MUS are discussed. TBMA is described and explanations provided of how TBMA has been specifically designed to support people’s insecure attachment styles. Three key concepts to support insecure attachment styles involved in the content of TBMA are identified and debated: (a) emotional regulation; (b) safety; and (c) bodymindfulness. There is a rationale for the design of TBMA as opposed to psychological interventions for this population. The programme’s structure, facilitation and content, takes account of the three insecure attachment styles above. Examples of how TBMA works with their specific characteristics are presented. TBMA has been tested and found to be effective during delivery in the United Kingdom National Health Service (NHS). Improved self-management has potential to reduce costs for the NHS and in General Practitioner time and resources.

## Introduction

This article builds on attachment theory (Bowlby, 1969; Holmes, 1993, 1994; Main, 2000; Holmes and Slade, 2018) and draws the links made between it and medically unexplained symptoms (MUS) by Adshead and Guthrie (2015). Its contribution to knowledge lies in that it describes how a novel group model, using a biopsychosocial perspective, called The BodyMind Approach^®^ (TBMA) (Payne, 2009a, b, 2015) supports people with MUS who also have insecure attachment. The rationale for the use of TBMA as opposed to psychological interventions is that the characteristics of insecure attachment are seen in some people with MUS so TBMA has been specifically designed in content and structure to work with these characteristics. It has been shown to be effective, in research, at reducing participant’s symptoms, anxiety and depression and increasing wellbeing, activity levels and overall functioning (Payne and Stott, 2010; Payne and Brooks, 2016, 2017, 2018; Payne, 2017a). The research also employed qualitative (participants comments, Payne and Brooks, unpublished) to assess the outcomes in an NHS community setting (Payne, 2014, 2017b). The concept here is that the effectiveness seen in the empirical research derives from the design (explained below in detail) of this novel approach which specifically addresses attachment-related issues for people suffering MUS. TBMA uses a learning treatment methodology with the aim of self-managing symptoms (Payne and Brooks, unpublished) rather than offering psychological treatment. We interpret self-management as an outcome due to the fact participants report seeking less external help for symptoms such as visiting General Practitioners (GPs), hospital and/or accident and emergency (A&E) departments. Therefore, TBMA provides a new, different and acceptable pathway for people with MUS and adds to the discourse and understanding of the condition and its management.

## Attachment

Attachment is the social connection that a child forms with a primary caregiver for emotional support/regulation (Munsell et al., 2012). Attachment happens during a “critical period” between six and twenty-four months enabling the child to create a working blueprint for future relationships. This forms an attachment style for the adult dependent on those from whom they seek and receive care (Bowlby, 1969), particularly relevant for people suffering MUS and seeking repeated care from the health service. Attachment style is embodied and to a large extent stored in implicit memory (Schachner et al., 2005; Bentzen, 2015).

When there is a perceived threat (real/imagined) to survival, wellbeing or safety, attachment behavior kicks-in to reduce distress for example, to increase proximity to, and receive soothing comfort/reassurance from, an identified attachment figure. Thereafter in the long term the adult has self-soothing behaviors for comfort when in distress, with healthy self-care and trust in the adequacy of caregivers.

## Medically Unexplained Symptoms

Medically unexplained symptoms are common world-wide affecting mostly women (Verhaak et al., 2006; Steinbrecher et al., 2011), young people and non-native speakers (Steinbrecher et al., 2011). Illness is the context from which their experience is constructed hence people with MUS tend to overly-identify with their symptoms. Research has found people with MUS have increased social isolation (Dirkzwager and Verhaak, 2007), more functional impairments (Katon and Walker, 1998), poorer quality of life (Smith et al., 1986), associated depression (Malhi et al., 2013), and anxiety (Lowe et al., 2008) when compared with non-MUS populations. Although moderate and severe MUS appear comorbidly with common mental disorders, a direct psychological causality to symptoms is too crude to explain most MUS (Henningsen et al., 2007).

One definition of MUS is chronic, persistent bodily symptoms for which no medical explanation has been found. MUS can also be termed “somatic symptom disorder” (SSD) (American Psychiatric Association, 2013) within the mental health field. It is defined as the total number of somatic symptoms and the degree to which the patient is concerned about them both of which are the predictors of health outcome and use.

Of the ten most common symptoms (fatigue, chest pain, headache, dizziness, swelling, back pain, insomnia, shortness of breath, abdominal pain, and numbness) GPs cannot find a medical explanation for 75% (Kroenke and Mangelsdorff, 1989). One in five GP consultations and 18% of consecutive attenders are for MUS (Taylor et al., 2012). Edwards et al. (2010) found studies from around the world showing MUS totals 26–35% in primary care and 50% in secondary care (Barsky and Borus, 1995).

Treatment studies have been varied with mixed outcomes. Most have been based on one single condition such as fibromyalgia, which has associated symptoms, although in practice patients have more than one additional condition. TBMA is different in that it can include all types of symptoms in one group. Schröder et al. (2012) is the only other approach which found group cognitive behavior therapy (CBT) to be effective with generic MUS conditions. TBMA is a group approach similar to those for specific symptoms in CBT (Arnold et al., 2004; Zonneveld et al., 2012) and group psychotherapy (for example Selders et al., 2015). Treatments are normally found in specialized clinics and mental health centers, limiting accessibility as patients refuse mental health referrals (Raine et al., 2002; Allen and Woolfolk, 2010). Approaches derived from individual CBT reduce the strength and occurrence of symptoms and improve functioning (den Boeft et al., 2014). Short-term intensive dynamic psychotherapy reduces symptoms and visits to A&E settings (Abbass et al., 2009). Mindfulness-based CBT may also be effective (van Ravesteijn et al., 2014). Training of GPs in reattribution therapy has had little success (Gask et al., 2011), however physical exercise (graded) and yoga have promising outcomes (Aamland et al., 2013; Yoshihara et al., 2014). None of the above mention insecure attachment styles.

## Attachment Issues

Not everyone has a secure attachment. Insecure attachment can derive from adverse child experiences (ACEs) such as neglect, emotional/physical/sexual abuse, separation, loss to create insecure future relationships (Murphy et al., 2014) into adulthood. The result is a vulnerability to manage stress, suppress negative feelings and care for self. Trust in the care-givers’ competence is eroded leading to withdrawal from help-seeking behavior (Ciechanowski et al., 2002) which may be true for some MUS sufferers, dependant on the insecure attachment style involved.

## Links Between Attachment Style and Medically Unexplained Symptoms

Bodily symptoms may be felt as a threat to survival, wellbeing and safety creating a susceptibility to an insecure attachment style. Adshead and Guthrie (2015) reviewed the evidence that insecure attachment is common in people with MUS and with some long-term conditions. They found three studies are relevant to insecure attachment style and MUS. For women in a health maintenance organization (Ciechanowski et al., 2002) only 34% had secure attachment which was half the expected number for a non-clinical sample. The women exhibited fearful (21%), pre-occupied (22%), and dismissing (23%) insecure attachment styles. Furthermore, the number of symptoms reported were significantly associated with these styles. A greater number of somatic symptoms were reported for preoccupied and fearful compared with secure. Attendance costs/call outs were higher for people with insecure attachment styles compared with secure. Patients presenting with MUS were 2.47 times more likely to have insecure attachment according to Taylor et al. (2000, 2012) showed frequent attendance at GPs was related to insecure attachment style.

Waller et al. (2004) assessed attachment security in 37 patients with ICD-10 somatoform disorder (without severe physical or mental illness) compared with 20 healthy matched controls. Compared with 60% of controls, only 26% rated as securely attached. The healthy controls demonstrated the expected incidence of insecure attachment, that is 25% were dismissing and 15% were pre-occupied. Patients though had high levels of dismissing (48.6%) and pre-occupied (25.7%) attachment styles in sharp contrast. Other studies showed how early insecure attachment styles are more common in patients with MUS (Taylor et al., 2000; Ciechanowski et al., 2002; Noyes et al., 2003; Spertus et al., 2003). It is proposed here that symptoms could be related to threats to attachment and thus to the self, resulting in fragility.

Using the natural stress adaptations e.g., flight, fight, (for mobilization) freeze, fold or faint (defensive immobilization) does not appear to resolve the internal perceived threat to wellbeing, survival and safety presented by MUS because the threat is in the body and not the environment. There is a correlation between female survivors of sexual abuse and preoccupied or insecure attachment (Stalker and Davies, 1995). Additionally, ACEs and somatization are linked (Waldinger et al., 2006), as are ACEs and attachment issues (Sansone et al., 2001). Insecure attachment has also been linked to somatization (Stuart and Noyes, 1999). Hence, ACEs are linked with both somatization (of which MUS is a subset) and attachment issues.

We know from research MUS is associated with cumulative ACEs, to include attachment issues (Elbers et al., 2017). We know also that insecure attachment creates stress and stress can result in mental health conditions and/or MUS. Thus, it could be concluded, having unexplained bodily symptoms might be a way for people with some insecure attachment styles to legitimately seek help to meet their physical needs from those expected to be unresponsive to emotional needs. Some insecure attachment styles result in the perception that health professionals are inadequate in reducing arousal levels to relieve stress. That is, the professional is experienced as the mirror of the early inadequate care-giver (i.e., the child’s primary care-giver).

## Hypothesis

Not everyone with MUS will experience insecure attachment. However, Adshead and Guthrie (2015) showed three insecure attachment styles are associated with MUS: dismissing, pre-occupied and fearful.

It has been demonstrated that TBMA is effective (Payne and Brooks, 2017) in promoting the self-management of symptoms. Building on the work of Adshead and Guthrie (2015), which demonstrates the link between MUS and some insecure attachment styles, TBMA has been specifically designed to take account of different insecure attachment styles. MUS presents as many and various symptoms. TBMA groups reflect this as they are heterogeneous. As a result, there will be some participants with insecure attachment as an underlying issue within these groups. At every stage, therefore, TBMA addresses issues of insecure attachment in the structure of the program, facilitation, group content/practices and mind-set of the population. Rather than one-to-one models, or non-interactive class-based methods, such as dance, Tai Chi or yoga, TBMA is a group interactive model. It supports people with MUS to take the risk of interacting with others (facilitator and other group members) within a safe, regulated environment. It may be that this interaction is the element of TBMA which helps address insecure attachment patterns.

We hypothesize to account for the effectiveness of TBMA, that it can address insecure attachment styles, which may be present in some MUS sufferers, leading to their capacity to self-manage. There are considerable benefits from TBMA as a specific type of bodymind approach that differs in that it is a group approach that avoids the stigma of, or aversion to, psychological therapies. In TBMA people learn to live well by self-managing symptoms. All this makes TBMA different from somatic therapies such as somatic experiencing (Levine, 2015), sensorimotor therapy (Ogden, 2006) and contemporary bodymind approaches. Whilst people report TBMA has helped them with their symptoms TBMA does not aim to transform trauma, relieve symptoms, help clients to discover the emotional and physical source of their trauma, discharge the consequences of that trauma from the nervous system, and then support their ability to self-regulate. Therefore, TBMA is unlike these models or any other psychological intervention.

The design of the model is apt for people with MUS because it is accessible and acceptable as a learning treatment methodology rather than a psychological treatment intervention. This population often do not accept or understand psychological methods/therapies due to their physical experiences and explanation for them. Consequently, TBMA can engage this hard-to-reach population.

## The Three Insecure Attachment Styles

Consequently, the three insecure attachment styles linked to MUS to which TBMA attends are: dismissing and pre-occupied (Bartholomew and Horowitz, 1991; Main, 2000); and fearful (Bartholomew and Horowitz, 1991). Not all participants attending TBMA groups will necessarily be insecurely attached, however, the program supports this population specifically and can be helpful to all.

### Dismissing

There may be an expectation that inadequate attention or care from others will be received with a “dismissing” type of attachment style. There may be anxiety about their symptoms and fear they will not be believed or taken seriously by health professionals. There may also be anxiety that the health professionals may assume there is a mental health condition. Therefore, any form of mental health referral is often rejected and generally the health service is seen as unhelpful. The GP and other health care providers may become, to the patient, “the inadequate clinician” as they attract the patient’s dismissive attitude.

### Pre-occupied

In contrast, an individual with a “pre-occupied” attachment style could become more concerned about losing the relationship with a health care professional after tests and scans etc., are over, and/or treatment is not indicated. There may be anxiety this relationship will need to end, they may become overly needy and dependent, pre-occupied with the relationship through their symptoms, so returning to the GP frequently. Bodily symptoms engage both parties, the patient visits the GP with more and more symptoms becoming emotionally needy of attention. The GP tries to find a resolution, so sends them again for more tests and scans etc., thus feeding their anxiety. These patients may be referred to by GPs as “frequent flyers.”

### Fearful

Waldinger et al. (2006) showed that fearful insecure attachment style is correlated with childhood ACEs and adult somatization in women. When a child is abused/neglected by a significant, yet unreliable adult caregiver, fearful attachment ensues. In this style a self-image may develop whereby the child feels unworthy of support from others, and of caregivers as being unreliable, or damaging. The combination of caregiver/GP and patient experience in the consultation may develop frustration and misunderstandings. Consequently, there may be a poor GP-patient relationship, and reduced care. The patient may feel they might drive others away and/or trigger inadequate outcomes due to their emotional neediness. Furthermore, this may develop into a compensatory emphasis on care-seeking for unexplained symptoms, due to an increased attention to bodily sensations.

## The Bodymind Approach^®^

We propose the insecure attachment above affect the sufferers’ ability to self-manage. Hence the need to develop a more secure attachment as part of learning to self-manage. TBMA appears to be effective for supporting people with MUS (Payne and Stott, 2010; Payne and Brooks, 2016, 2017, 2018, unpublished), and we suggest this as a result of increasing secure attachment, in some participants, enabling the development of self-management. Due to TBMA’s purpose-built design (discussed in detail below) insecure attachment may be re-worked. In our experience, working with the symptoms through the body using improvisation, movement play, clay modeling, collage, mark-making, bodymindfulness, creativity and body-mind-emotion connections enable participants to explore and access meaning (Kossak, 2009). Using the imagination and creativity in movement, for example, can tap into sensory-emotional connections allowing embodied tacit knowledge of the symptom (which may otherwise be inaccessible) to surface. In contrast to CBT, TBMA uses the notion of the embodied unconscious (van der Kolk, 2014) by accessing the sensory experience in the body acquired through lived experience of the symptom. Accessing meaning explicitly invites people to make their own interpretations of the symptoms, for example when making marks or moving hands to describe how they feel about/experience their symptom. This symbolizes for themselves their unconscious meaning of the symptom which helps to make their previously unconscious experience explicit, similar to how arts therapies work. However, the authors are unaware of any arts therapies being employed for supporting people with MUS to self-manage. Establishing meaning helps the participant to validate the symptom. This is liberating because many MUS sufferers have been disbelieved.

The embodied style of attachment will be symbolized by the relationship to the symptom. Cognitive behavior therapy comes at the world from thinking about thinking (meta-cognition) i.e., content. TBMA, in contrast, when employing bodymindfulness comes at the world from the awareness of awareness (meta-witness of the experience of sensation and process). The ability to have awareness of awareness enables people to recognize the possibility of non-attachment to the symptom (Wallin, 2017).

Adshead and Guthrie (2015) propose mindfulness-based practices may help with MUS by improving regulation of negative affect and to alter the awareness of, and relationship to, pain and bodily experience. Additionally, they suggest approaches offering “here and now” bodily experience connecting with images whereby links can emerge between physical sensations, emotions and relationships. They go on to recommend that “clinicians need to develop interventions that “fit” the attachment narratives of individual patients, rather than forcing patients into one size fits all psychological therapeutic techniques” (Adshead and Guthrie, 2015: 8). TBMA satisfies this recommendation because it has been specifically designed to fit the attachment narratives of individuals, additionally in a group setting. Furthermore, TBMA works with the imagination and bodily experiences, and somatic mindfulness practices to help people make connections between emotions, sensations and relationships. TBMA works in the “present moment” to raise and change awareness of the bodily sensation and the individual’s relationship to it (Payne, 2019). TBMA is framed as experiential learning (Kolb, 1984; Payne and Brooks, unpublished) as well as transformative in adult learning (Payne et al., 2019). The exercises enable access to perceptions of symptoms through the facilitator coaching enactive embodied mindful practices. They aim to shift the experience of the symptom, changing the relationship, perception and mind-set toward the symptom. This leads to the cultivation of self-management of symptoms thereby encouraging wellbeing.

Unlike psychological interventions in TBMA the body is emphasized first and foremost hence bodymind, joined together, rather than “mind-body” with “mind” written first and separated from “body” with a hyphen. TBMA works from the subjective body experience to the mind and back again. It privileges the interactive relationship between the body and mind, which is so emphasized in MUS. TBMA is focused holistically on the whole person rather than relying solely on language with more of a focus on the right side of the brain (creative side). In TBMA there is no explicit discussion of psychological or causal relationship with the symptoms unless the participant makes such connections themselves.

The BodyMind Approach^®^ transforms seeing symptoms or the body as the “enemy” in a dismissive attachment style to embracing them as an “ally” flagging up the need for self-care and compassionate acceptance of symptoms/self (Payne and Brooks, unpublished). Caring for the self (self-soothing normally developed from early attachment experiences) is initially modeled by the facilitator as a proxy caregiver e.g., how to sit, breathe, use bodymindfulness and listen to the body for signs of stress. Practices compare symptom sensations with other areas of the body as functioning and positive to create a balance between health and “dys-ease.” Rather than immobility, as often found in mindfulness, TBMA encourages mindful mobility/mindful movement which favors agency, and somatic mindfulness, for example, “being in the movement moment” as in walking around the space together with a focus on what is happening in the body and to the symptom in action.

Group interaction is important to aid different styles of attachment with peers rather than solely with the facilitator, who for some may be a health professional to whom they may have a corresponding negative attitude (dismissive style). This attitude may not be so prominent with the group members. The group gives the opportunity for shared resources, a sense of belonging helps engagement, reduces isolation and promotes hormones to be released, for example, dopamine, oxytocin, serotonin, and endorphins (Porges, 2003; van der Kolk, 2014).

## Three Key Concepts

The BodyMind Approach^®^ is designed to support people with MUS and insecure attachment to learn to self-manage through three key concepts pragmatically built into the program.

(a) Emotional regulation;
(b) Safety;
(c) Bodymindfulness.

### Emotional Regulation

Emotional regulation is how a person manages feelings with cognitive, physiological and behavioral associated processes. It is the process that raises or lowers the degree of emotions (Parrott, 1993) to enhance wellbeing. This emotional self-regulation framework provides for vitality but also reduced arousal for calmness. It is developed through attunement with a reliable caregiver. Attachment is therefore a significant aspect of emotional self-regulation. More securely attached children rate higher in emotional regulation and empathy (Panfile and Laible, 2012). TBMA appears to overcome the powerful blueprint of early insecure attachment, using the relationship with the facilitator and the group to cultivate a more secure relationship enabling the development of resilience drawing on neuroplasticity.

Holmes (1993) reporting on Bowlby indicates that attachment is a primary motivational system related to a spatial environment in association with a loved one. When an individual feels safe and securely attached to the loved one they can begin to pursue exploration. When they feel unsafe dysregulated signs of distress appear in behavior. TBMA engages with individuals to explore their symptoms by providing a safe environment. The facilitator models unconditional positive regard and a non-judgmental attitude. When this is combined with stable closed group membership (few withdrawals), a constant space, predicted dates/times for meetings and a consistent facilitator, safety ensues making for regulated behavior.

### The Importance of Safety in Groups

Participants were requested to commit for the first six sessions and thereafter for the following six. The opportunity to withdraw after the first six sessions appeared to add to the safety element for some people but was never used. Paradoxically it seems likely that this structure was less threatening for individuals with a fearful or dismissive style enabling them to complete the 12 sessions. Participants with a pre-occupied style would feel compelled to complete anyway.

In Maslow’s (1943) hierarchy of needs for self-actualization the first is physiological then comes safety needs followed by the need for a sense of belonging. Insecure attachment means that a sense of belonging is missing, maybe because social engagement is too difficult. We know reliable safety is crucial to allow social engagement to occur. When safety and wellbeing is threatened, as in MUS, there is a greater need for safety to reduce the activation of the stress adaption response of mobility (Porges, 2018). In people with both MUS and insecure attachment the need for safety is even more critical. Hence the group needs to be a safe place, non-threatening and social to give a sense of belonging through the shared purpose. Another aspect of safety in TBMA sessions is that no one need disclose their symptom/s which helps enable experimentation and exploration of symptoms.

### Bodymindfulness

Depression and/or anxiety often accompany MUS (Rosmalen and de Jonge, 2010; Burton et al., 2011). Mindfulness reduces depression and anxiety (Hofmann et al., 2010) and has a moderate effect on some MUS, such as pain (Grossman et al., 2004). Segal et al. (2002) found an association between a lack of mindful self-awareness and depression, resulting in poor recognition of, and reflection on, bodily cues or signals like tension, pain, fatigue. A “mindful attitude” can be defined as a state of presence moment to moment, realized through intentionally directed attention. At the same time both internal body sensations and external stimuli can enter and leave awareness without judgment. For example, in kindly attending to the symptom sensation interoceptively can, ironically, reduce the distress experienced. A mindful state results from participating in this state as though one was an empathic witness “benignly regarding the self.”

“Bodymindfulness” incorporates body awareness practices and movement in the present moment (“kinesthetic mindfulness”). It can help with dis-identification with bodily symptoms which is so often tied up with identity for the individual with MUS (Sanders et al., 2018).

## The Design of TBMA to Support Insecure Attachment

The intervention is referred to as “learning groups”; “symptoms groups” and “workshops” with a focus on the lived body experience of the symptoms rather than any mental health or psychological title. People are referred to as “participants” rather than “patients” which may help a sense of agency since it reduces dependency and any expectations the facilitator will be unsatisfactory. The program normalizes the symptoms, i.e., non-medicalizing them which helps acceptance of the condition and promotes feelings of agency, where previously there may have been none. For all insecure attachment styles this sense of agency can be helpful for engagement.

The group workshops are held twice a week for the first 2 weeks. This intensity at the outset helps to promote cohesiveness in the group. Bonds can be forged with each other and the facilitator, promoting engagement and reducing drop-out. The 12 × 2 hourly sessions are optimal for change (Lambert, 2013) with enough time for engagement. The individual consultation with the facilitator conducted before the group commences and the week it ends is in the same venue as the group sessions which can add reassurance for individuals with pre-occupied insecure attachment styles. Participants are aware they will be contacted by text, email and letter by the facilitator every 6 weeks for a further 6 months, i.e., they are not dropped after the group ends. A participant who has a pre-occupied insecure attachment style will be reassured by the level of contact on-going, initially the fearfully attached will be frightened but they can opt in or out after six sessions. The participant with a dismissive insecure attachment style will disengage and sabotage the group. However, the facilitator having a very high level of psychological skills can “hold” the group and provide enough safety to prevent disintegration occurring.

The sessions are carefully structured to cultivate interaction with rituals and predictable events for safety which will have supported participants with fearful or preoccupied insecure attachment styles substantially. There is predictable on-going contact between participants and facilitator, even after face-to-face contact has concluded, via text, email and letters, seems to reduce concerns whether participants have fearful, dismissing or a pre-occupied attachment style.

### The Power of the Group

For people with MUS who are insecurely attached the group can act as a support and pathway toward learning to make healthy attachments in a safe setting. The group acts as a source of peer support rather than support being from one health professional i.e., from only the therapist/teacher as in one-to-one approaches. Friendships test out and strengthen the ability to form more secure attachments. Group solidarity and approbation develop, encouraging each other toward improvement. The group shares goals, for example, improving health and wellbeing and the belief in hope for change. These shared goals/beliefs help form the group identity, rationale for the sense of belonging, the protection offered, and the group’s continuous existence through the bond created (Bar-Tal, 2000). This type of group for this population which have tended to have experienced isolation can be a welcome “comfort blanket” bridging them into a different world of experimentation and exploration.

The group gives permission to share intimate personal stories. Participants discover common experiences shared in the group, they feel less isolated, make friends and often meet up following the group. The fact people wish to meet after the group is in line with group identification and group attachment. Smith et al. (1999) explain the subsystems and functions regulating one-to-one attachment are the same as attachment to social groups. These include seeking support and responsiveness and emotional disclosure, all of which are affected by personal history which in turn affects future relationships. Bearing this in mind careful preparation is given to the beginning and ending of sessions and of the whole program. For example, cohesion is strongly encouraged, and safety promoted from the outset. Additionally, there are individual consultations with the facilitator, an action plan for going forward post-group and non-face-to-face contact every 6 weeks for 6 months. The group’s capacity to act as an attachment object and provider of security can affect neural integration. The group may help to down-regulate participants’ emotions by being a regular, steady influence in their lives. Porges’ Polyvagal Theory (Porges, 2003) concludes that human social interaction combined with taking the psychological mind-set into account in interventions turns off the sympathetic fight/flight response. The calming of the sympathetic nervous system, combined with feeling listened to, enables people to feel safe enough to engage in the play. This enables the work of creativity, imagination, self-reflection, self-regulation and self-management (Porges, 2003).

It is possible that the group may be self-selecting since people who tend to avoid attachment or who are anxiously attached may filter themselves out before committing. Anxiously attached participants may be frightened of rejection so might be overly positive of their experiences.

### The Facilitator as a Catalyst

Bowlby (1982: 207) suggests “the link between leader and group is a facilitating, rather than a necessary element of the individual’s attachment to the group.” Sochos (2015) claims there can be an attachment to the group via an image which symbolizes the group. There is a sense of security and protection derived from the leader - a powerful other - however, in TBMA the attachment is with the facilitator and group members. It is symptom which can be symbolized.

The facilitator initially holds the hope for the group and that change is possible which helps transform the group mind-set to a more positive one. Facilitators have a passion for the approach which influences engagement from the group. They are all trained and certified in TBMA, have experience of over 5 years in leading groups of adults in mental health and a background in embodied, enactive approaches. Furthermore, facilitators are selected based on their qualities of warmth, empathy, and genuineness (Rogers, 1961). The facilitator’s training and attitudes are specifically geared toward supporting individuals with insecure attachment.

The individual consultation with the group facilitator at the outset sets the tone for the group workshops, building early rapport with the group facilitator to provide safety. An insecurely attached participant will have opportunities to see and experience secure attached relationships, and to transform the relationship with the facilitator over time. This early relationship set up may help calm anxieties and helps to ensure future participation and relationship formation.

The individual consultation with the facilitator at the end of the group helps reflection, closure, clarification of their action plan and support arranged for this during the following 6 months. This session provided for preparation for the ending of the group face-to-face is so important for pre-occupied insecurely attached participants who will not have had many experiences of good-enough endings. The subsequent 6 months of non-face to face contact with the facilitator supports continuity, a sense of agency to self-manage and the embedment of new habits promoted via their action plan.

Each insecure attachment style has its own characteristics and we speculate on how these are interacted with through the design of structure, facilitation and practices of the TBMA intervention below.

### Dismissive

In this style there is a positive view of self (I am ok) and a negative view of others (you are not ok). A dismissing type of attachment style may bring the expectation of inadequate attention or care will be received from others. Those who care for them, such as GPs are not OK. In TBMA people are in a group with shared experiences of the health service which may, perhaps, reinforce their lived experience of inadequate care. However, the other participants are not their health professionals (not authority figures) and this is an important advantage for their sources of support. People share their experience, strengths and hopes for change. This is empowering. Participants are encouraged to consider ways to care for themselves (self-sooth), manage stress levels and re-interpret their symptom distress.

This individual usually rejects any form of mental health referral and generally sees the health service as unhelpful for their MUS. In order to facilitate acceptance and access for this style TBMA is framed as “workshops” for “self-management” rather than a medical intervention or mental health treatment methodology.

People with a dismissive style deny and minimize the impact of their own experience and their feelings. They tend to lack confidence in the helper and in their ability to help themselves. They may have poor self-reflection and tend to be critical of practices and helpers to date (e.g., GP). In order to accommodate this the facilitator accepts and welcomes their stance non-judgmentally and reflects it back to the participant to support and validate it. This avoids criticism of the helper. Other group members then act as models for reflection, again taking their attention away from the facilitator. The facilitator encourages mobilization to generate more experience on which to reflect and to think about the meaning of their symptom.

### Pre-occupied

In the pre-occupied style people tend to feel overwhelmed by their symptoms. The stance taken by the facilitator is that many people have unexplained symptoms which she can work with thus normalizing the condition reducing fear. There is also the threat of what will happen if they lose their symptoms i.e., a leap of faith into the unknown. Eventually, after a while, when trust has been established this can be addressed by exploring the pros and cons of having the symptom. The facilitator forms a stable attachment figure, as does the group thus engendering trust. The non-verbal communication of the body is a root to access what is unknown, as yet, regarding the meaning of the symptom. So, practices employing movement such as gestures and postures to represent the sensation of the symptom may bring meaning to the forefront and in-depth knowing which cannot be arrived at in any other way.

In the pre-occupied attachment style, there is a negative model of self, a positive model of others- “I am not OK, others are OK.” The pre-determined frequency and nature of the contact post group is reassuring for people with a pre-occupied insecure attachment style. The facilitator models self-acceptance and compassion enabling people to develop a more solid, coherent sense of self and to acknowledge their own vulnerabilities resulting from their experiences.

Additionally, since the attachment style is more secure as a result of the TBMA program this may enable them to become less dependent on the GP as the monitoring of the 6 months follow up data showed. This participant may find the ending of the group problematic and experience it as loss. The closing meeting with the facilitator mitigates some of this but also groups do tend to go on voluntarily meeting up following the ending. Another, strategy to support the participant who has a pre-occupied insecure attachment style is the on-going non-face to face contact every 6 weeks post group. The shared-decision making (with the facilitator) of their tailor-made action plan (derived from experiences in the group to support new habits of self-management) also helps with the ending process and sustainability.

The efficacy of TBMA in promoting self-management enables participants who have a pre-occupied attachment style to accept their condition obviating the need for further tests and scans. TBMA promotes a belief they can live well and thrive despite their symptoms. Their symptom distress levels and anxiety decrease as they let go of the need for a medical explanation.

### Fearful

Individuals who have a fearfully insecure attachment style have a negative model of self and others - neither are OK. They may present as angry, frustrated, difficult, prone to develop a self-image as unworthy of support from others and of caregivers as unreliable, or even dangerous. TBMA promotes a sense of agency and self–care i.e., deserving of care for themselves. The facilitator understands the importance of always present for the group demonstrating reliability, which in turn offers safety. Both participants with fearful insecure attachments and the facilitator may experience misunderstanding and frustration. However, regular supervision supports the facilitator to contain any frustrations and to ensure best practice when working with this participant.

People with a fearful insecure attachment style may worry about not being believed and/or taken seriously by health care providers who may assume they have a mental health condition. In TBMA the participant’s lived body experience is believed and symptoms honored. They also worry about their symptoms which defy diagnosis despite numerous tests and scans which can lead to catastrophizing about them. The embodied, pre-verbal feelings, thoughts, relationships and impulses form an attachment style in childhood which is repeated symbolically in the adult’s relationship to their symptom. TBMA helps people change their stance toward their experience of the symptom through a shift in the view of self. This may be a dynamic relationship with the symptom and the self. The view of self becomes much more than simply the symptom thus reducing the tendency to catastrophize.

The participant may sense their emotional neediness may drive others away. Emotional needs are welcome in the group, although the facilitator ensures shared attention is available to each member. People who are fearfully attached may avoid long-term care situations because of concerns about greater intimacy with providers and an assumption they will be given insufficient care. Hence TBMA is short term, the number of sessions overall is 12, the first four are in the first 2 weeks (i.e., two sessions per week), of 2 h duration each, with an opt-out after session six. Twelve sessions are the optimum for engagement for group psychotherapy according to Lambert (2013).

Fears about caregiver dependability promotes GP-shopping, i.e., visiting each GP in a practice and/or changing practices frequently, and a fragmentation of care. TBMA groups have a number of participants to offer resources and care. The caregiver may experience people who are fearfully attached as difficult to reassure, inadequate, needy, and fragile. Facilitators are trained to expect participants like this and have strategies to support them e.g., offering alternatives to practices, treating the practices as experiments to try out – reducing risk and stakes, lessening exposure. Individual consultations with the facilitator before the group sessions provide an opportunity for this participant to ask questions and gain reassurance leading to feelings of safety. This mediates the initial stress of attending a group of unknown people.

The outreach of 6 months non-face-to-face contact subsequent to the group ending can feel safer than being in the group whilst maintaining an on-going relationship with the group facilitator. This can replace seeking care in settings such as A&E. TBMA is designed to support participants over a period of 9-month from acceptance of the referral. It has been found that the 12 face-to-face sessions over 10 weeks in the first 3 months are just about manageable and bearable for the participant who is fearfully insecurely attached.

## Conclusion

The research conducted previously supports the hypothesis that TBMA can support people with insecure attachment styles and MUS to self-manage. This article has illustrated how the design of TBMA is built on three insecure attachment styles associated with MUS. It goes on to explain how TBMA helps people with MUS and insecure attachment styles to learn to self-manage. Its contribution to knowledge lies in that it describes a novel group model TBMA designed specifically as a new alternative pathway for supporting people with MUS, some of whom may be insecurely attached. TBMA is particularly suited as an intervention for people with MUS because symptoms are experienced in the body first and foremost. TBMA honors those symptoms using them as a gateway to the mind and subsequent self-management, in contrast to CBT which tend to marginalize the body. TBMA is also different because it is a groupwork model including people with all sorts of conditions in a generic group.

Early attachment is first experienced through the body via touch from the primary caregiver (White, 2004). Body memory (Giuseppe, 2018) of early attachment is reflected in relationships in the future, including the relationship with the symptom which can become a metaphor for the individual’s insecure attachment. TBMA works with the symptom and its meaning employing the body-felt sensation of the symptom as the basis for learning self-management. It seems likely that the pain from ACEs is transported into the body unconsciously and held there as a bodily memory only to be triggered in response to stressful situations to form a MUS. By learning to address the stress MUS suffering can be self-managed.

The BodyMind Approach^®^ is innovative since all elements involved have been designed to compensate for insecure attachment issues. This includes program structure, qualities of facilitation, group methods and content to take account of safety, self-regulation, and bodymindfulness. The group and facilitator are crucial to outcomes for participants helping them to prevent the repetition of a dysfunctional attachment style, affecting the maintenance of self-management to sustain recovery. TBMA enables a re-sculpting of the self and the symptom and their relationship to each other. The improved self-management participants exhibit when tested for effectiveness through practice-based evidence resulted in reduced symptom distress, depression, anxiety and increased wellbeing, activity and overall functioning. It is proposed the behavior changes noted have become conscious which is essential for self-management. Importantly, there are also potential reduced costs for the health service and in GP time and resources (Payne, 2014).

The hypothesis that TBMA can address insecure attachment in people with MUS can be tested in the framework of current knowledge by conducting an adult attachment assessment (Bartholomew and Shaver, 1998) pre and post intervention with participants suffering MUS undergoing TBMA treatment.

## Author Contributions

HP researched the literature and developed the TBMA program. SB contributed equally to the writing of the manuscript.

## Conflict of Interest Statement

The authors declare that the research was conducted in the absence of any commercial or financial relationships that could be construed as a potential conflict of interest.

## References

[B1] Aamland A. Werner E. L. Malterud K. (2013). Sickness absence, marginality, and medically unexplained physical symptoms: a focus-group study of patients’ experiences. *Scand. J. Prim. Health Care* 31 95–100. 10.3109/02813432.2013.788274 23659708 PMC3656402

[B2] Abbass A. Campbell S. Magee K. Tarzwell R. (2009). Intensive short-term dynamic psychotherapy to reduce rates of emergency department return visits for patients with medically unexplained symptoms: preliminary evidence from a pre-post intervention study. *CJEM* 11 529–534. 10.1017/s1481803500011799 19922712

[B3] Adshead G. Guthrie E. (2015). The role of attachment in medically unexplained symptoms and long-term illness. *Br. J. Psychol. Adv.* 3 167–174. 10.1192/apt.bp.114.013045

[B4] Allen L. A. Woolfolk R. L. (2010). Cognitive behavioral therapy for somatoform disorders. *Psychiatr. Clin. N. Am.* 33 579–593. 10.1016/j.psc.2010.04.014 20599134

[B5] American Psychiatric Association (2013). *Diagnostic and Statistical Manual of Mental Disorders-5.* Washington, DC: American Psychiatric Association.

[B6] Arnold I. A. Speckens A. E. M. Van Hermert A. M. (2004). Medically unexplained physical symptoms: the feasibility of group cognitive-behavioural therapy in primary care. *J. Psychosom. Res.* 57 517–520. 15596157 10.1016/j.jpsychores.2004.04.369

[B7] Barsky A. J. Borus J. F. (1995). Somatization and medicalization in the era of managed care. *JAMA* 274 1931–1934. 10.1001/jama.274.24.1931 8568987

[B8] Bar-Tal D. (2000). *Shared Beliefs in a Society: Social Psychological Alysis*. London: Sage.

[B9] Bartholomew K. Horowitz L. M. (1991). Attachment styles among young adults: a test of a four-category model. *J. Pers. Soc. Psychol.* 61 226–244. 10.1037//0022-3514.61.2.226 1920064

[B10] Bartholomew K. Shaver P. R. (1998). “Methods of assessing adult attachment,” in *Attachment Theory and Close Relationships*, eds Simpson J. A. Rholes W. S. (New York, NY: Guilford Press).

[B11] Bentzen M. (2015). Dances of connection: neuro-affective development in clinical work with attachment. *Body Mov. Dance Psychother.* 10 211–226. 10.1080/17432979.2015.1064479

[B12] Bowlby J. (1969). *Attachment and Loss.* New York, NY: Basic Books.

[B13] Bowlby J. (1982). *Attachment*, 2nd Edn New York, NY: Basic Books.

[B14] Burton C. McGorm K. Weller D. Sharpe M. (2011). Depression and anxiety in patients repeatedly referred to secondary care with medically unexplained symptoms: a case-control study. *Psychol. Med.* 41 555–563. 10.1017/S0033291710001017 21272387

[B15] Ciechanowski P. S. Walker E. A. Katon W. J. Russo J. E. (2002). Attachment theory: a model for health care utilization and somatization. *Psychosom. Med.* 64 660–667. 10.1097/01.psy.0000021948.90613.76 12140356

[B16] den Boeft M. van der Wouden J. C. Rydell-Lexmond T. R. de Wit N. J. van der Horst H. E. Numans M. E. (2014). Identifying patients with medically unexplained physical symptoms in electronic medical records in primary care: a validation study. *BMC Fam. Pract.* 515:109. 10.1186/1471-2296-15-109 24903850 PMC4052805

[B17] Dirkzwager A. J. E. Verhaak P. F. M. (2007). Patients with persistent medically unexplained symptoms in general practice: characteristics and quality of care. *BMC Fam. Pract.* 8:33. 10.1186/1471-2296-8-33 17540013 PMC1894968

[B18] Edwards T. M. Stern A. Clarke D. D. Ivbijaro G. Kasney L. M. (2010). The treatment of patients with medically unexplained symptoms in primary care: a review of the literature. *Ment. Health Fam. Med.* 7 209–221. 22477945 PMC3083260

[B19] Elbers J. Rovnaghi C. R. Golianu B. Anand K. J. S. (2017). Clinical profile associated with adverse childhood experiences: the advent of nervous system dysregulation. *Children* 4:98. 10.3390/children4110098 29140276 PMC5704132

[B20] Gask L. Dowrick C. Salmon P. Peters S. Morriss R. (2011). Reattribution reconsidered: narrative review and reflections on an educational intervention for medically unexplained symptoms in primary care settings. *Psychosom. Res.* 71 325–334. 10.1016/j.jpsychores.2011.05.008 21999976

[B21] Giuseppe R. (2018). The neuroscience of body memory: from the self through the space to the others. *Cortex* 104 241–260. 10.1016/j.cortex.2017.07.013 28826604

[B22] Grossman P. Niemann L. Schmidt S. Walach H. (2004). Mindfulness-based stress reduction and health benefits. A meta-analysis. *J. Psychosom. Res.* 57 35–43. 15256293 10.1016/S0022-3999(03)00573-7

[B23] Henningsen P. Zipfel S. Herzog W. (2007). Management of functional somatic syndromes. *Lancet* 369 946–955. 17368156 10.1016/S0140-6736(07)60159-7

[B24] Hofmann S. G. Sawyer A. T. Witt A. A. Oh D. (2010). The effect of mindfulness-based therapy on anxiety and depression: a meta-analytic review. *J. Consult. Clin. Psychol.* 78 169–183. 10.1037/a0018555 20350028 PMC2848393

[B25] Holmes J. (1993). *John Bowlby and Attachment Theory.* London: Routledge.

[B26] Holmes J. (1994). Attachment theory - a secure theoretical base for counselling? *Psychodyn. Couns.* 1 65–78. 10.1080/13533339408404713

[B27] Holmes J. Slade A. (2018). *Attachment in Therapeutic Practice.* Thousand Oaks, CA: Sage.

[B28] Katon W. J. Walker E. A. (1998). Medically unexplained symptoms in primary care. *J. Clin. Psychiatr.* 59 15–21. 9881537

[B29] Kolb D. A. (1984). *Experiential Learning: Experience as the Source of Learning and Development.* Englewood Cliffs, NJ: Prentice-Hall.

[B30] Kossak M. S. (2009). Therapeutic attunement: a transpersonal view of expressive arts therapy. *Arts Psychother.* 36 13–18. 10.1016/j.aip.2008.09.003

[B31] Kroenke K. Mangelsdorff A. D. (1989). Common symptoms in ambulatory care: incidence, evaluation, therapy & outcome. *Am. J. Med.* 86 262–266. 10.1016/0002-9343(89)90293-3 2919607

[B32] Lambert M. J. (2013). “The efficacy and effectiveness of psychotherapy,” in *Handbook of Psychotherapy and Behaviour Change*, eds Bergin A. E. Garfield S. L. (Hoboken, NJ: John Wiley and Sons).

[B33] Levine P. (2015). *Trauma and Memory: Brain and Body in a Search for the Living Past: A Practical Guide for Understanding and Working with Traumatic Memory.* Berkeley, CA: North Atlantic Books.

[B34] Lowe B. Spitzer R. L. Williams J. B. Mussell M. Schellberg D. Kroenke K. (2008). Depression, anxiety and somatization in primary care: syndrome overlap and functional impairment. *Gen. Hosp. Psychiatry* 30 191–199. 10.1016/j.genhosppsych.2008.01.001 18433651

[B35] Main M. (2000). The adult attachment interview: fear, attention, safety and discourse processes. *J. Am. Psychoanal. Assoc.* 48 1055–1095. 11212183 10.1177/00030651000480041801

[B36] Malhi G. S. Couston C. M. Fizt K. (2013). Unlocking the diagnosis of depression in primary care: which key symptoms are gps using to determine diagnosis and severity? *Aust. N. Z. J. Psychiatry* 48:6. 10.1177/0004867413513342 24270311

[B37] Maslow A. H. (1943). A theory of human motivation. *Psychol. Rev.* 50 370–396. 10.1037/h0054346

[B38] Munsell E. P. Kilmer R. P. Cook J. R. Reeve C. L. (2012). The effects of caregiver social connections on caregiver, child, and family well-being. *Am. J. Orthopsychiatry* 82 137–145. 10.1111/j.1939-0025.2011.01129.x 22239404 PMC3345204

[B39] Murphy A. Steele M. Dube S. R. Bate J. Bonuck K. Meissner P. (2014). Adverse childhood experiences (aces) questionnaire and adult attachment interview (aai): implications for parent child relationships. *Child Abuse Negl.* 38 224–233. 10.1016/j.chiabu.2013.09.004 24670331

[B40] Noyes R. J. Stuart S. P. Langbehn D. R. Happel R. L. Longley S. L. Muller B. A. (2003). Test of an interpersonal model of hypochondriasis. *Psychosom. Med.* 65 292–300. 10.1097/01.psy.0000058377.50240.64 12651997

[B41] Ogden P. (2006). *Trauma and the Body: A Sensorimotor Approach to Psychotherapy* (Norton Series on Interpersonal Neurobiology). New York, NY: Norton.

[B42] Panfile T. M. Laible D. J. (2012). Attachment security and child’s empathy: the mediating role of emotion regulation. *Merrill Palmer Q.* 58 1–21. 10.1353/mpq.2012.0003

[B43] Parrott W. G. (1993). “Beyond hedonism: motives for inhibiting good moods and for maintaining bad moods,” in *Handbook of Mental Control*, eds Wegner D. M. Pennebaker J. W. (Englewood Cliffs, NJ: Prentice Hall).

[B44] Payne H. (2009a). Pilot study to evaluate dance movement psychotherapy (the bodymind approach) with patients with medically unexplained symptoms: participant and facilitator perceptions and a summary discussion. *Int. J. Body Mov. Dance Psychother.* 5 95–106.

[B45] Payne H. (2009b). The BodyMind Approach to psychotherapeutic groupwork with patients with medically unexplained symptoms: a review of the literature, description of approach and methodology selected for a pilot study. *Eur. J. Couns. Psychother.* 11 287–310. 10.1080/13642530903230392

[B46] Payne H. (2014). Patient experience: push past symptom mysteries. *Health Serv. J.* 124 26–27. 24933838

[B47] Payne H. (2015). The Body speaks its mind: the bodymind approach^®^ for patients with medically unexplained symptoms in UK primary care. *Arts Psychother.* 42 19–27. 10.1016/j.aip.2014.12.011

[B48] Payne H. (2017a). “Reliable change in outcomes from the bodymind approach^®^ with people who have medically unexplained symptoms/somatic symptom disorder in primary care,” in *Essentials in Dance Movement Psychotherapy: International Perspectives on Theory Research and Practice*, ed. Payne H. (Abingdon: Routledge). 10.1016/j.aip.2014.12.011

[B49] Payne H. (2017b). Transferring research from a university into the National Health Service: implications for impact. *Health Res. Syst. Policy* 15:56. 10.1186/s12961-017-0219-3 28623933 PMC5473996

[B50] Payne H. (2019). “The bodymind approach^®^ and people affected by medically unexplained symptoms/somatic symptom disorder,” in *The Routledge International Handbook on Embodied Perspectives in Psychotherapy*, eds Payne H. Koch S. Tantia S. J. Fuchs T. (Abingdon: Routledge).

[B51] Payne H. Brooks S. (2016). Clinical outcomes and cost benefits from the bodymind approach^®^ for patients with medically unexplained symptoms in primary health care in england: Practice-based evidence. *Arts Psychother.* 47 55–65. 10.1016/j.aip.2015.12.001

[B52] Payne H. Brooks S. (2017). Moving on: the bodymind approach for medically unexplained symptoms. *J. Public Ment. Health* 16 1–9.

[B53] Payne H. Brooks S. (2018). Different strokes for different folks: the bodymind approach^®^ as a learning tool for patients with medically unexplained symptoms to self-manage. *Front. Psychol.* 9:2222. 10.3389/fpsyg.2018.02222 30483203 PMC6243086

[B54] Payne H. Jarvis J. Roberts A. (2019). The bodymind approach^®^ as transformative learning to promote self-management for patients with medically unexplained symptoms. *J. Trans. Educ.* (in press).

[B55] Payne H. Stott D. (2010). Change in the moving bodymind: quantitative results from a pilot study on the Bodymind Approach (BMA) as groupwork for patients with medically unexplained symptoms (MUS). *Couns. Psychother. Res.* 10 295–307.

[B56] Porges S. (2003). Social engagement and attachment: a phylogenetic perspective. *Ann. N.Y. Acad. Sci.* 1008 31–47. 14998870 10.1196/annals.1301.004

[B57] Porges S. (2018). *Clinical Applications of Polyvagal Theory: The Emergence of Polyvagal Informed Therapies.* New York, NY: WW Norton and Co.

[B58] Raine R. Haines A. Sensky T. Hutchings A. Larkin K. Black N. (2002). Systematic review of mental health interventions for patients with common somatic symptoms: can research evidence from secondary care be extrapolated to primary care? *Br. Med. J.* 325 1082–1092. 12424170 10.1136/bmj.325.7372.1082 PMC131187

[B59] Rogers C. (1961). *On Becoming a Person.* London: Constable.

[B60] Rosmalen J. G. M. de Jonge P. (2010). Empirical foundation for the diagnosis of somatization: implications for DSM-5. *Psychol. Med.* 41 1133–1142. 10.1017/S0033291710001625 20843407

[B61] Sanders T. Winter D. Payne H. (2018). Personal constructs of mind-body identity in people who experience medically unexplained symptoms. *J. Constr. Psychol.* 1–16. 10.1080/10720537.2018.1515047

[B62] Sansone R. A. Wiederman M. Sansone L. (2001). Adult somatic preoccupation and its relationship to childhood trauma. *Violence Vict.* 16 39–47. 10.1891/0886-6708.16.1.39 11281223

[B63] Schachner P. R. Shaver M. Mikulincer M. (2005). Patterns of nonverbal behavior and sensitivity in the context of attachment relationships. *J. Nonverbal Behav.* 3 141–169. 10.1007/s10919-005-4847-x

[B64] Schröder A. Rehfeld E. Ornbol E. Sharpe M. Licht R. W. Fink P. (2012). Cognitive-behavioural group treatment for a range of functional somatic syndromes: randomised trial. *Br. J. Psychiatry* 200 499–507. 10.1192/bjp.bp.111.098681 22539780

[B65] Segal Z. V. Williams J. M. G. Teasdale J. D. (2002). *Mindfulness-Based Cognitive Therapy for Depression: A New Approach to Preventing Relapse.* New York, NY: Guilford.

[B66] Selders M. Visser R. van Rooij W. Delfstra G. Koelen J. A. (2015). The development of a brief group intervention (Dynamic Interpersonal Therapy) for patients with medically unexplained somatic symptoms: a pilot study. *Psychoanal. Psychother.* 29 182–198. 10.1080/02668734.2015.1036106

[B67] Smith E. Murphy J. Coats S. (1999). Attachment to groups: theory and management. *J. Pers. Soc. Psychol.* 77 94–110. 10.1037//0022-3514.77.1.94 10434410

[B68] Smith G. R. Monson R. A. Ray D. C. (1986). Patients with multiple unexplained symptoms: their characteristics, functional health, and health care utilization. *Arch. Intern. Med.* 146 69–72. 10.1001/archinte.146.1.69 3942467

[B69] Sochos A. (2015). Beyond interpersonal relationships: attachment to social groups, ideological systems, and social institutions. *Psychologist* 28 986–991.

[B70] Spertus I. L. Yehuda R. Wong C. M. Halligan S. Seremetis S. V. (2003). Childhood emotional abuse and neglect as predictors of psychological and physical symptoms in women presenting to a primary care practice. *Child Abuse Negl.* 27 1247–1258. 10.1016/j.chiabu.2003.05.001 14637300

[B71] Stalker C. A. Davies F. (1995). Attachment organization and adaptation in sexually abused women. *Can. J. Psychiatry* 40 234–240. 10.1177/070674379504000503 7553541

[B72] Steinbrecher N. Koerber S. Frieser D. Hiller W. (2011). The prevalence of medically unexplained symptoms in primary care. *Psychosomatic* 52 263–271. 10.1016/j.psym.2011.01.007 21565598

[B73] Stuart S. Noyes R. (1999). Attachment and interpersonal communication in somatization. *Psychosomatics* 40 34–43. 10.1016/s0033-3182(99)71269-7 9989119

[B74] Taylor R. E. Mann A. H. White N. J. Goldberg D. P. (2000). Attachment style in patients with unexplained physical complaints. *Psychol. Med.* 30 931–941. 10.1017/s0033291799002317 11037101

[B75] Taylor R. E. Marshall T. Mann A. Goldberg D. P. (2012). Insecure attachment and frequent attendance in primary care: a longitudinal cohort study of medically unexplained symptom presentations in ten UK general practices. *Psychol. Med.* 4 855–864. 10.1017/S0033291711001589 21880165

[B76] van der Kolk B. (2014). *The Body Keeps the Score: Mind, Brain and Body in the Transformation of Trauma.* London: Penguin Books.

[B77] van Ravesteijn H. J. Suijkerbuijk Y. B. Langbroek J. A. Muskens E. Lucassen P. L. van Weel C. (2014). Mindfulness-based cognitive therapy (MBCT) for patients with medically unexplained symptoms: process of change. *Psychosom. Res.* 77 27–33. 10.1016/j.jpsychores.2014.04.010 24913338

[B78] Verhaak P. F. Meijer S. A. Visser A. P. Wolters G. (2006). Persistent presentation of medically unexplained symptoms in general practice. *Fam. Pract.* 23 414–420. 10.1093/fampra/cml016 16632487

[B79] Waldinger R. J. Schulz M. S. Barsky A. J. Ahern D. K. (2006). Mapping the road from childhood trauma to adult somatization: the role of attachment. *Psychosom. Med.* 68 129–135. 10.1097/01.psy.0000195834.37094.a4 16449423

[B80] Waller E. Scheidt C. E. Hartmann A. (2004). Attachment representation and illness behavior in somatoform disorders. *J. Nervous Ment. Dis.* 192 200–209. 10.1097/01.nmd.0000116463.17588.07 15091301

[B81] Wallin D. J. (2017). *Attachment in psychotherapy.* New York, NY: Guilford Press.

[B82] White K. (2004). *Touch: Attachment and the body.* London: Karnac.

[B83] Yoshihara K. Hiramoto T. Oka T. Kubo C. Sudo N. (2014). Effect of 12 weeks of yoga training on the somatization, psychological symptoms, and stress-related biomarkers of healthy women. *Biopsychosoc. Med.* 8:1. 10.1186/1751-0759-8-1 24383884 PMC3892034

[B84] Zonneveld L. N. L. van Rood Y. R. Timman R. Kooiman C. G. Spijker A. Busschbach J. J. V. (2012). Effective group training for patients with unexplained physical symptoms: a randomized controlled trial with a non-randomized one-year follow-up. *PLoS One* 7:e42629. 10.1371/journal.pone.0042629 22880056 PMC3413637

